# Climate warming may affect the optimal timing of reproduction for migratory geese differently in the low and high Arctic

**DOI:** 10.1007/s00442-019-04533-7

**Published:** 2019-10-17

**Authors:** Thomas K. Lameris, Margje E. de Jong, Michiel P. Boom, Henk P. van der Jeugd, Konstantin E. Litvin, Maarten J. J. E. Loonen, Bart A. Nolet, Jouke Prop

**Affiliations:** 1 Department of Animal Ecology, Netherlands Institute of Ecology (NIOO-KNAW), Wageningen, The Netherlands; 2 Theoretical and Computational Ecology, University of Amsterdam, Amsterdam, The Netherlands; 3 Arctic Centre, University of Groningen, Groningen, The Netherlands; 4 Vogeltrekstation-Dutch Centre for Avian Migration and Demography (NIOO-KNAW), Wageningen, The Netherlands; 5 Bird Ringing Centre of Russia, IEE RAS, Moscow, Russia; 6 Present Address: Department of Behavioural Biology, University of Vienna, Vienna, Austria; 7 Present Address: NIOZ Royal Netherlands Institute for Sea Research, and Utrecht University, Den Burg, The Netherlands

**Keywords:** Barnacle goose, *Branta leucopsis*, Trade-off, Fitness, Phenology

## Abstract

Rapid climate warming is driving organisms to advance timing of reproduction with earlier springs, but the rate of advancement shows large variation, even among populations of the same species. In this study, we investigated how the rate of advancement in timing of reproduction with a warming climate varies for barnacle goose (*Branta leucopsis*) populations breeding at different latitudes in the Arctic. We hypothesized that populations breeding further North are generally more time constrained and, therefore, produce clutches earlier relative to the onset of spring than southern populations. Therefore, with increasing temperatures and a progressive relief of time constraint, we expected latitudinal differences to decrease. For the years 2000–2016, we determined the onset of spring from snow cover data derived from satellite images, and compiled data on egg laying date and reproductive performance in one low-Arctic and two high-Arctic sites. As expected, high-Arctic geese laid their eggs earlier relative to snowmelt than low-Arctic geese. Contrary to expectations, advancement in laying dates was similar in high- and low-Arctic colonies, at a rate of 27% of the advance in date of snowmelt. Although advancement of egg laying did not fully compensate for the advancement of snowmelt, geese laying eggs at intermediate dates in the low Arctic were the most successful breeders. In the high Arctic, however, early nesting geese were the most successful breeders, suggesting that high-Arctic geese have not advanced their laying dates sufficiently to earlier springs. This indicates that high-Arctic geese especially are vulnerable to negative effects of climate warming.

**Electronic supplementary material:**

The online version of this article (10.1007/s00442-019-04533-7) contains supplementary material, which is available to authorized users.

## Introduction

The earth’s climate has warmed rapidly in the past decades, resulting in warmer and earlier springs (Schwartz et al. 2006; Stocker et al. 2013). In response, many migratory bird species have advanced their arrival on the breeding grounds (Jonzén et al. 2006; Gunnarsson and Tómasson 2011; Gill et al. 2014) as well as the dates at which they lay their eggs (Crick et al. 1997; Pearce-Higgins et al. 2005; Gill et al. 2014). A general finding is that this advance does not fully compensate for any forward shifts of seasonal peaks in food abundance (Thackeray et al. 2010; Gienapp et al. 2014). This inability to advance timing of reproduction may lead to a so-called phenological mismatch between the peak in food availability and the moment of high energy requirements of the growing young (Both and Visser 2005), with potential negative consequences for fitness (Both et al. 2006; Visser et al. 2012; Doiron et al. 2015). At the same time, an advancement of the breeding season may diminish nutritional stress for the parents (Boyd and Madsen 1997) and relieve seasonal time constraints on the birds’ reproductive cycle (Tomotani et al. 2016) with potential positive effects on reproductive output (Dickey et al. 2008; Van Oudenhove et al. 2014; Lameris et al. 2017b).

To what extent birds should advance their laying dates under climate warming revolves around a trade-off between the most favourable conditions for the parent bird versus the most favourable conditions for its offspring (Lack 1968; Trivers 1974). This trade-off is especially important in strongly seasonal environments with a short annual breeding season (Tomotani et al. 2016, 2018) such as the Arctic, where migratory birds are on a tight schedule to raise offspring and prepare for their return journey to the wintering grounds. While benefitting from favourable conditions for the hatched offspring requires early laying dates to avoid a phenological mismatch, parents may also benefit from laying their eggs later (Perrins 1970; Drent 2006). For example, for Arctic-nesting geese, it is known that postponing egg laying allows for more time to obtain body stores, which enables birds to lay more eggs (Rowe et al. 1994), and leads to more favourable conditions for foraging during the incubation period (Prop and de Vries 1993; Eichhorn et al. 2010). As larger body stores and better foraging conditions enable birds to have shorter incubation recesses (Aldrich and Raveling 1983; Tombre et al. 2012) and thereby reduce the chance of nest predation (Prop et al. 1984), postponing egg laying adds to the probability of successfully hatching the clutch (Prop and de Vries 1993). The outcome of the decision when to produce a clutch may depend on the factor that is most strongly limiting reproductive output, and may thus vary depending on the environmental conditions, including climate.

Since the Arctic breeding season is restricted to the short snow-free summer, fitness is most likely limited by the time available for the offspring to become full-fledged (Owen 1980), and birds should start laying eggs as soon as body stores allow (Prop and de Vries 1993). Within the Arctic, this will hold even stronger for birds breeding in the high Arctic, where snow cover is more prolonged than in the low Arctic. A warming climate may relieve a time constraint (Gaston et al. 2005), as food becomes available earlier (Lameris et al. 2017a) and birds are able to collect more local food resources prior to laying (Hupp et al. 2018). In this way, earlier springs can have positive effects on reproductive output via increased nesting propensity (Syroechkovskiy et al. 1991; Madsen et al. 2007; Dickey et al. 2008), clutch size (Rowe et al. 1994; Van Oudenhove et al. 2014), and nesting success (Prop and de Vries 1993). However, birds may only be able to tune laying dates to an earlier phenology of the food if they are not constrained by the timing of arrival on the breeding grounds (Both and Visser 2001; Lameris et al. 2018). This may form a particular important constraint for birds breeding at higher latitudes in the Arctic, given their longer migratory journeys (Drent and Piersma 1990) and inability to predict climatic conditions on the breeding grounds (Tombre et al. 2008; Kölzsch et al. 2015).

**Fig. 1:**
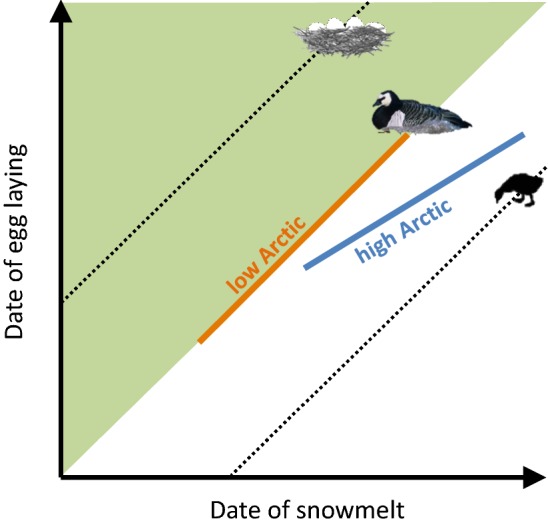
Graphical model exploring how laying dates could vary with climatic conditions at lower and higher latitudes. Earlier date of snowmelt (as a proxy for food phenology; white–green transition) advance the phenology of required conditions for clutch survival (upper dotted lines) and for the hatched offspring (lower dotted lines), which together drive the timing of egg laying. Laying dates are thought to vary by latitude within the Arctic. Birds at in the low Arctic (orange line) lay their eggs close to the date of snowmelt and advance laying dates in synchrony with earlier dates of snowmelt. Birds in the high Arctic (blue line) face shorter breeding seasons and, therefore, lay their eggs earlier relative to the date of snowmelt. Constrained by conditions during migration birds adjust laying dates at a lower rate than the advance in date of snowmelt (note the difference in slope between the orange and blue line) (color figure online)

A graphical model serves to illustrate how climatic conditions impinge on laying dates in the low and the high Arctic (Fig. 1). This is exemplified by Arctic-nesting geese, which adjust their laying dates to date of snowmelt (Prop and de Vries 1993; Bêty et al. 2003; Madsen et al. 2007). The date of snowmelt can be used as a measure of food phenology on the breeding grounds, since the peak in food availability is linked to the date of snowmelt (Tulp and Schekkerman 2008; Lameris et al. 2018), and is a main driver of fitness (Barry 1962; Lameris et al. 2018). Decisions on when to produce a clutch then result from a trade-off between laying early, i.e. before the date of snowmelt, to leave prime conditions for the hatched offspring, and postponing laying to after the date of snowmelt to benefit from prime conditions to increase clutch size and enhance clutch survival. Given that breeding seasons towards the North are shorter (Owen 1980; Klaassen et al. 2006) and moreover, birds are likely more constrained by conditions encountered en route, while migrating towards their breeding grounds, we propose the following three hypotheses. (1) In the low Arctic, birds will lay their eggs close to the date of snowmelt, and adjust laying dates with a changing date of snowmelt to achieve optimal conditions for their offspring (orange line with a slope of 1 in Fig. 1). (2) In the high Arctic, birds must lay their eggs earlier relative to the date of snowmelt due to the shorter breeding season (blue solid line in Fig. 1). (3) In the high Arctic, birds adjust laying dates at a lower rate than the variation in date of snowmelt as they are likely constrained by arrival on the breeding grounds, which results in a slope of the relationship between dates of laying and snowmelt of less than 1 (blue solid line in Fig. 1).

Here, we ask whether a long-distance migratory bird species breeding in the low and the high Arctic adjusts timing of reproduction optimally to climate warming, by testing the above-described hypotheses. For this purpose, we collated data from the years 2000–2016 on dates of egg laying and reproductive output in barnacle geese (*Branta leucopsis)* from three different study populations at low- and high-Arctic sites. To understand whether the observed laying dates were optimal or resulted from a constraint (following hypothesis 3), we explored whether reproductive output peaked at intermediate laying dates, or whether geese that produced their eggs earlier gained a higher reproductive success. We determined the optimal timing of breeding by examining laying-date-specific reproductive output, focusing on the period between arrival at the breeding grounds and the moment of gosling hatch.

## Methods

### Study sites

**Fig. 2:**
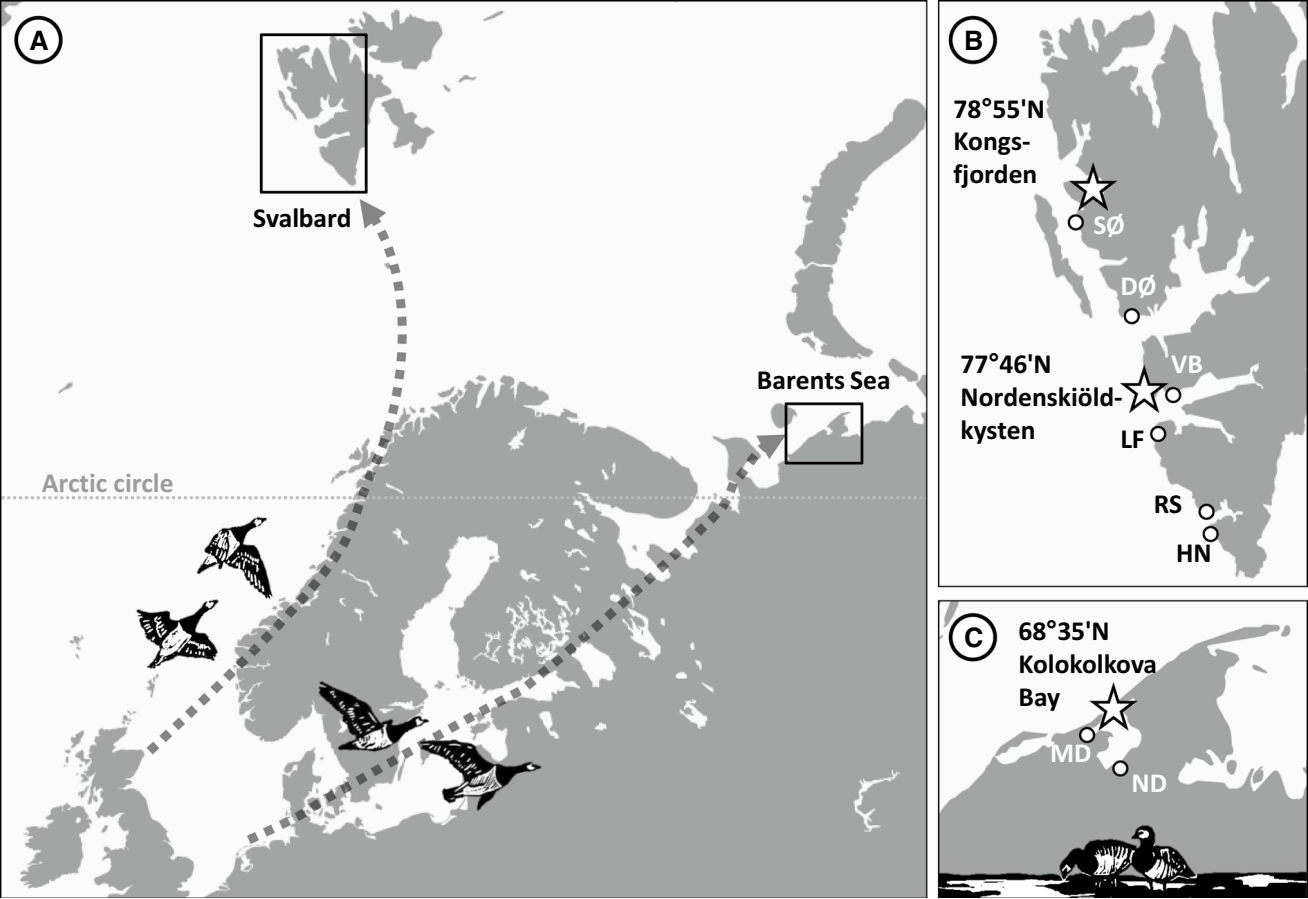
The flyways of barnacle geese breeding on Svalbard and along the Barents Sea coast (**a**) with location of study colonies (stars) and staging sites (white circles) in the high Arctic on Svalbard (**b**) and in the low Arctic at the Barents Sea coast (**c**). Dotted arrows sketch migration routes. Staging site names are abbreviated: Barents Sea (*ND* Neruta river delta, *MD* Molotsnii river delta); Svalbard (*HN* Hornsundneset, *RS* Ralstrånda, *LF* Lognedalsflya, *VB* Vårsolbukta, *DØ* Daudmannsøyra, *SØ* Sarsøyra)

**Fig. 3:**
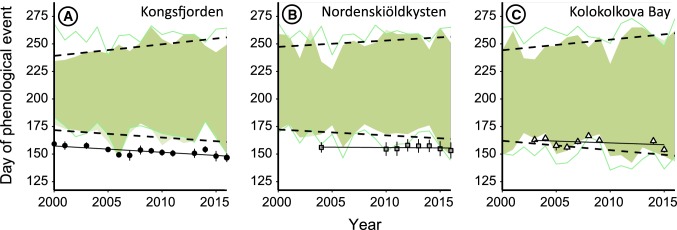
Annual snow-free periods (indicated by green area) for barnacle geese in the high Arctic (**a** Kongsfjorden and **b** Nordenskiöldkysten) and in the low Arctic (**c** Kolokolkova Bay) during the years 2000–2016. Linear trends over the years of dates of snowmelt and snowfall are indicated by dashed black lines. Dates of snowmelt and snowfall at the staging sites are indicated by green lines. Average laying dates are indicated by symbols and associated error bars (showing standard deviations), and linear trends are indicated by solid black lines (color figure online)

Arctic-nesting barnacle geese are divided into three flyway populations, with geese breeding in Eastern Greenland, Svalbard and along the Barents Sea coast; wintering in Ireland, the UK and the Netherlands/Germany, respectively (Madsen et al. 1999, Fig. 2a). Between 2000 and 2016, barnacle geese were studied in three breeding colonies, of which two are located in the high Arctic (Svalbard) and one in the low Arctic (at the Russian coast of the Barents Sea). (1) On the islet Storholmen in Kongsfjorden (KF), Svalbard (78°55′N, 12°12′E, Fig. 2b); (2) on the islet Diabasøya and adjacent tundra at Nordenskiöldkysten (NSK), Svalbard (77°46′N, 13°42′E, Fig. 2b) and (3) surrounding the abandoned village of Tobseda at the Kolokolkova Bay (KB), Russia (68°35′N, 52°20′E, Fig. 2c). While these sites vastly differ in geographical position, they are all lowland sites in close proximity to the coast, which facilitates comparison. Data collection in the colonies took place in different years: Kongsfjorden (2000, 2001, 2003, 2005–2016); Nordenskiöldkysten (2004, 2010–2016), Kolokolkova Bay (2003–2009, 2014, 2015). From geographic positions of geese equipped with tracking devices (Tombre et al. 2017; Lameris et al. 2018), we determined staging sites close to the breeding grounds, which geese use prior to moving to the breeding colonies. Geese forage at these staging sites until conditions become suitable for laying in the breeding colonies (Hübner 2006; Lameris et al. 2018). We identified three proximate staging sites, which were closest to the breeding colonies (Hübner 2006), on Svalbard: Lognedalsflya (LF), Vårsolbukta (VB), Sarsøyra (SØ), (Fig. 2b); and two sites around the Kolokolkova Bay: Neruta river delta (ND) and Molotsnii river delta (MD) (Fig. 2c). We further identified another three southern staging sites on Svalbard, which were at a larger distance from the colonies on Svalbard: Hornsundneset (HN), Ralstrånda (RS), Daudmannsøyra (DØ).

### Snow cover

We used the period between the moment of snowmelt (spring) and snowfall (autumn) as a measure of the length of the Arctic plant growth season. Snowmelt is an important driver of the timing of reproduction (Madsen et al. 2007) through its effect on the phenology of Arctic plants (Prop and de Vries 1993; Livensperger et al. 2016). Snowfall in autumn, on the other hand, puts a rigid end to feeding opportunities and thus to the breeding season. We estimated daily snow cover (as percentage cover over total area) for all study sites and years (see Table S1 for an overview of sites) for the period with sufficient daylight (26 February–30 September) using satellite images of the MODIS snow cover product (MOD10a2 version 6, Hall et al. 2006). As the areas for the breeding colonies KF and NSK were too small to determine snow cover, we instead chose the nearest coastal tundra which geese used for foraging during egg laying (Prop and de Vries 1993). To limit the effects of clouds obscuring the image, composite satellite images were generated over 8 days. Any composite images with a cloud cover exceeding 25% were excluded. A pixel (500 m resolution) was assigned as snow when classified as snow at least once during an 8-day period. With a spatial overlay of the breeding areas with the MODIS images, the number of pixels classified as snow within the study site could be retrieved. From the number of snow pixels and the total number of pixels in the breeding area, we calculated the percentage of snow cover. We linearly interpolated between values from composite images to attain a daily percentage of snow cover. From the snow cover data, we extracted the date of snowmelt, which we defined as the first day of the season at which snow cover was less than 50% (a measure which correlates with date of peak food quality; Lameris et al. 2018). Similarly, we calculated the moment of snowfall as the last day of the season at which snow cover was less than 30%. We chose this cut-off value for snowfall as higher levels of snow cover were not always reached before 30 September. We calculated season length at the breeding colony as the interval between dates of snowmelt and snowfall.

### Nesting parameters

Per nest, we determined egg-laying date (date when first egg was laid), clutch size (number of eggs laid), number of hatchlings (number of eggs which successfully hatched) and nest success (whether a nest produced hatchlings) for as many nests as possible (Table S2). The parameters and the precise methodologies varied among breeding colonies. We collected data on egg-laying dates, number of hatchlings and nest fate in all colonies, and data on clutch size in KB and KF colonies. In addition, we recorded the number of nests in the colonies.

In the KB colony, we systematically searched for nests and checked nests every 2–3 days during the laying and early incubation period (late May–late June). Eggs were marked and the number of eggs was recorded at every visit. In the early and mid-incubation period, we determined clutch size as the total number of eggs in a nest when encountered with the same number of eggs during two subsequent visits. We excluded nests in which egg dumping was evident (more eggs per interval than expected or additional eggs after clutch completion). We visited nests during hatch (mid-June–late July) every 2 days to estimate date of hatch and record nesting success and number of hatchlings. We recorded nest fate as successful, predated, flooded or abandoned. A nest was considered successful when at least one chick had hatched, which we determined either by presence of hatchlings at the nest or presence of egg membranes and trampled nest rim (Davis et al. 1998). Empty nests and nests containing eggshells without membranes were considered as predated, or considered flooded when the nest was partly under water. Nests encountered after the laying period containing cold eggs and without nest owners present were considered abandoned. We recorded the number of hatchlings when (1) at least 50% of the eggs were in the process of hatching (cracks, hatching or hatched chicks), or (2) less than 50% of the eggs had successfully hatched (thus goslings present) and other eggs did not show signs of hatching. For the number of hatchlings, we assumed all eggs with signs of hatching to produce hatchlings. Hatching success was calculated as the number of eggs hatched divided by clutch size. To minimize disturbance, not all successful nests were visited at hatch. The total number of nests found in the colony during the study period was recorded as a measure of nesting propensity.

In the KF colony, the same methods were applied, except that nests were only visited from the early incubation period onwards, and not in the laying period.

At the NSK colony, we observed the goose colony on an offshore island from an observation tower on the mainland, 200 m away from the colony. Nests were monitored 6–16 h/day during the period that nesting geese were present. Nests in view of the tower were mapped on high-resolution images of the island, which enabled us to assess the breeding history of individual birds by visual observation from laying until hatching. 30–60% of the pairs was recognizable by coded leg rings (either one or both partners carrying a ring). As we did not find a difference in any of the parameters estimated between marked and unmarked pairs, all pairs were used in subsequent analyses. To avoid disturbance, the island was not visited during the breeding period and, therefore, clutch sizes were not determined. Nest fate was established from direct observations, and rated as successful (at least one gosling was seen at the nest and no predation of eggs or goslings was observed), predated (eggs or chicks were taken by a predator, most often polar bears *Ursus maritimus*), or abandoned (nest owners abandoned the nest territory before the eggs hatched and prior to any predation event taking place—after which the eggs were usually taken by glaucous gull *Larus hyperboreus*). The number of hatchlings was recorded by visual observation of nests that successfully hatched. The first day that goslings were seen at the nest rim was taken as the date of hatch. The total number of nests was recorded for every year.

For KB and NSK, we used nest fate to calculate the nesting success as the proportion of initiated nests that successfully hatched per year. As nests were not observed during the entire incubation period in the KF colony, we did not calculate nesting success for KF. To combine nesting success and number of hatchlings into a single measure of reproductive success, we calculated the total number of expected hatchlings per nest, per laying date, year and colony, as the product of (1) nesting success and (2) average number of hatchlings in successful nests.

### Laying dates

Methods to determine the date of egg laying differed among study sites. In KB, laying date was estimated by back calculation for clutches found during egg laying, assuming a laying interval of 33 h, as follows: day of discovery when one egg was found; day of discovery minus 1 at two eggs; day of discovery minus 3 at three eggs; day of discovery minus 4 at four eggs (van der Jeugd et al. 2009). Both in KB and KF, laying dates were also back calculated from hatch date. Hatch date was estimated for clutches found in the process of hatching as follows: date of observation was taken when the nest contained at least one egg with holes, a hatching chick or a wet chick; 1 day was subtracted from the date of observation when all chicks were fluffy and dry; 1 day was added to the date when the nest contained only eggs with cracks. For back calculation, we assumed a period of 29 days between laying date and hatch date (as derived from 573 nests in the KB colony between 2005 and 2015 for which both lay and hatch date were determined), which is similar to results from NSK (30 days between laying date and hatch date, derived from 99 nests for 2010–2016). In NSK, laying date was estimated as the first day during which a pair occupied a territory. Territories that were occupied for only 1 day were not considered in analyses.

Site-specific approaches in collecting data might affect the potential to make comparisons between study sites. In KB and NSK, where we used back-calculated as well as observed laying dates, the close proximity of the period between laying dates and hatch dates (see above) gives us reason to believe that these methods are comparable. By back calculating laying dates from hatching dates as done for the KF colony, we did not take into account the laying dates of nests which did not survive until hatch. However, we found no reason to suspect that this affected estimates of laying dates considerably, as extensive nest searches in the colony throughout the incubation period indicated that only few nests were lost (7.5% on average).

### Statistics

We tested relationships between date of snowmelt, laying dates and reproductive success by linear models in R 3.5.1 (R Development Core Team 2018), using the package “lme4” (Bates et al. 2018). We added year and/or study site (all sites where we measured snow cover) as random factors to account for either the different years during which data were collected when a trend over years was not of interest, or to account for data from different study sites when the specific sites were not of interest. Candidate models were constructed from all possible combinations of predictor variables, including interactions which were considered ecologically meaningful. All models were compared using Akaike’s information criterion corrected for small sample sizes (AICc; Burnham and Anderson 2004) and we chose the model with the lowest AICc value as our final model. Models within 2 ΔAICc of the final model were considered as competitive as long as these did not contain extra, potentially uninformative, parameters in comparison to the final model (Arnold 2010). Model-averaged parameter estimates were obtained by the package MuMln (Bartoń 2018). Support of the selected model (or models) relative to next best model was calculated from the ratio of model weights (Burnham et al. 2011). Besides predictor variables relating to snow cover, study year and the fitness components (clutch size, number of hatchlings and nesting success), we used predictor variables which separated high- and low-Arctic sites and staging and breeding sites, including ‘area’ (high or low Arctic), ‘site’ (all sites from which we gathered data on snow cover), ‘site type’ (southern staging sites/proximate staging sites/breeding colonies) and ‘colonies’ (the three study colonies).

First, to analyse if the snow-free period differed between the high and the low Arctic and among years, we ran linear mixed effect models (LMMs) with date of snowmelt/snowfall/season length as a response variable, year as fixed factor and area as fixed covariate, with site as a random factor. To test whether the snow-free period differed between breeding and staging sites, we ran LMMs with date of snowmelt as a response variable and site type and area as fixed factors, and with site and year as random factors.

Second, to analyse whether laying dates differed among years and between colonies, we used a linear regression model (LM) with average yearly laying dates as a response variable, and year and colony as fixed factors. To analyse how laying dates were affected by date of snowmelt, we ran LMMs with yearly average laying date as a response variable, date of snowmelt (in colonies and at proximate staging sites) and colony as fixed effects, and year as random factor. We tested whether the difference between laying dates and date of snowmelt at proximate staging sites differed between colonies by running an LMM with the difference in days as a response variable, colony as a fixed factor, and year as random factor.

Third, we aimed to analyse the association between laying date and date of snowmelt with fitness components. We ran generalized linear regression models (GLMs) with a Poisson distribution for clutch size and number of hatchlings as response variables, and GLMs with a binomial distribution with a logit link function for hatching success as a response variable. In these GLMs, we included colony as fixed factor and either date of snowmelt or laying date as fixed covariates. We tested the effects of these variables in separate models as the variables were highly correlated. A year effect was accounted for by date of snowmelt, and therefore, year was not included in the analyses as an additional covariate. We tested the association between clutch size and number of hatchlings in an LMM with year and site as random factors. Furthermore, we ran GLMs with a binomial distribution and a logit link function with nesting success as response variable. We included either laying date and laying date squared or date of snowmelt as predictor variables. For NSK, we excluded the years 2012 and 2014, when nest success was 0. We ran LMMs with total expected number of hatchlings as response variable, included laying date and laying date squared as response variables. We ran a similar analysis for total expected number of hatchlings in a GLM per year to retrieve slopes per year per site. We tested the association between number of nests and date of snowmelt in an LM, including colony as a fixed factor.

## Results

### Snowmelt and snowfall

In the high Arctic, snow melted 16 ± 2 (SD) days later than in the low Arctic (19 June and 3 June in high and low Arctic, respectively, Fig. 3), and between 2000 and 2016 the date of snowmelt advanced at similar rates in the high and low Arctic by on average 0.66 ± 0.12 days/year (Table S7A; model without interaction term year and area is 3.0 times more likely than model with interaction term, Table S4A). Snow in colonies melted 4.37 ± 1.66 days later compared to the date of melt at proximate staging sites, similar in the high and low Arctic (Table S7B; model with interaction term area and site type contains more parameters, and model without is 1.4 times more likely than model with interaction term, Table S4A). As a result of earlier spring snowmelt and later autumn snowfall, the snow-free period became longer by on average 1.06 ± 1.20 days per year (Fig. 3 and Table S7D; S4D), and was 13–16 days longer in the low Arctic as compared to the high Arctic (Table S3).

### Timing of reproduction

**Fig. 4:**
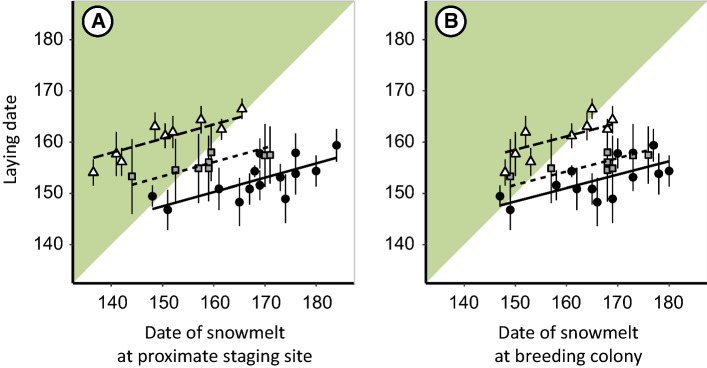
Relationship between yearly average laying dates (shown with standard deviations) and date of snowmelt at proximate staging sites (**a**) and in breeding colonies (**b**). Lines show linear regressions, symbols and line types correspond to the three study sites: Kongsfjorden (black dots, solid lines), Nordenskiöldkysten (grey squares, dotted lines) and Kolokolkova Bay (white triangles, dashed lines)

Laying date was inversely related to latitude, with barnacle geese in the high-Arctic sites laying the earliest, and geese in the low Arctic laying up to 12 days later (3 June in KF, 5 June in NSK, 10 June in KB, Figs. 3, 4). Geese advanced their egg-laying dates at a rate of 0.43 ± 0.12 days per year which did not differ between colonies (model without interaction between colony and year is 5.6 times more likely than model without interaction term, Table S5A). Laying dates were positively related to date of snowmelt at proximate staging sites (regression coefficient 0.27 ± 0.05, Fig. 4a and Table S8B) and in colonies (regression coefficient 0.26 ± 0.05, Fig. 4b and Table S8B). This relationship did not differ between colonies (model with interaction term snowmelt at proximate staging sites and year contains more parameters than model without interaction term and model without is 1.8 times more likely than model with interaction term, Table S5B). Models containing date of snowmelt at proximate staging sites gained higher support than models containing date of snowmelt in colonies (models 1 is 70.1 times more likely than model 5, Table S5B). Therefore, we performed subsequent analyses with snowmelt data of proximate staging sites. Geese in the high Arctic produced eggs on average before the date of snowmelt, while low-Arctic geese laid their eggs after the date of snowmelt (KF: 16 ± 8 days before date of snowmelt, NSK: 3 ± 7 days before date of snowmelt, KB: 10 ± 6 days after date of snowmelt, Fig. 4a, Table S8B). The number of nests was not associated with date of snowmelt (model without date of snowmelt is 4.3 times more likely than model with, Table S6A).

### Clutch size

**Fig. 5:**
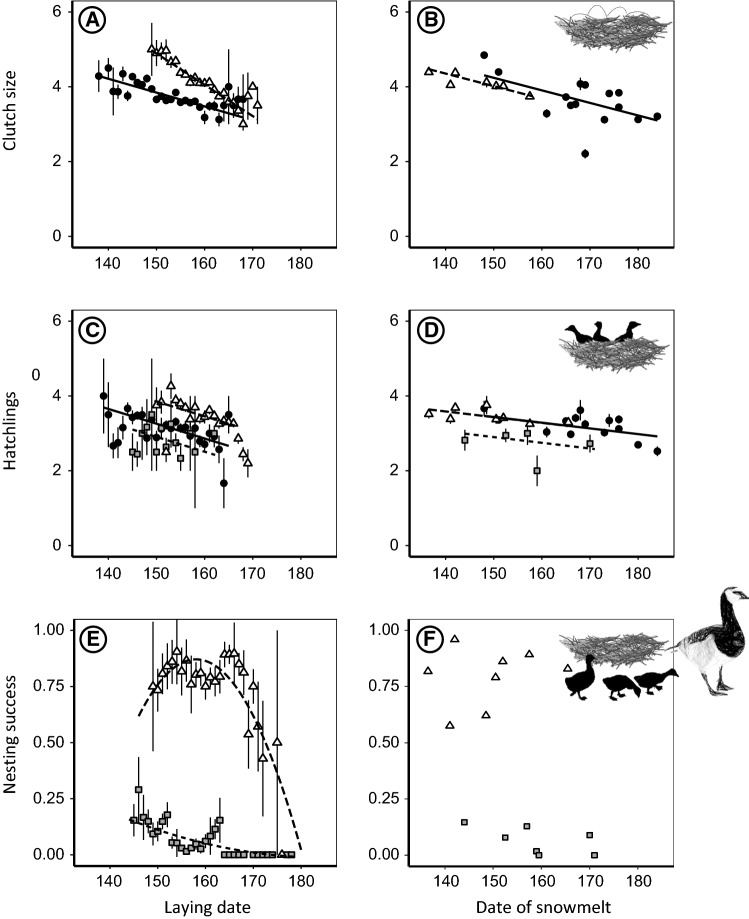
Clutch size (**a**, **b**), number of hatchlings (**c**, **d**) and nesting success (**e**, **f**) in relation to laying date (**a**, **c**, **e**) and date of snowmelt at proximate staging sites (**b**, **d**, **f**). Estimates are the averages by laying date across years (**a**, **c**, **e**), and by year (**b**, **d**, **f**), with error bars depicting standard errors. Lines show linear regressions resulting from model averaging, symbols correspond to the three study sites: Kongsfjorden (black dots, solid lines), Nordenskiöldkysten (grey squares, dotted lines) and Kolokolkova Bay (light grey triangles, dashed lines). In E, nesting success is depicted for KB for all years, and for NSK, only for years when nesting success was higher than 0

Clutch size declined with laying date with a steeper seasonal decline in the low Arctic than in the high Arctic (KF: regression coefficient = − 0.037 ± 0.008 egg per day; KB: regression coefficient = − 0.084 ± 0.020 egg per day, Fig. 5a and Table S9B; model with interaction term colony and laying date is 99.0 times more likely than model without interaction term, Table S6B). Clutch size also declined with date of snowmelt, at similar rates in the high and low Arctic (regression coefficient = − 0.034 ± 0.004 egg per day, Fig. 5b and Table S9C; model with interaction term colony and date of snowmelt contains more parameters than model without interaction term and model without is 2.0 times more likely than model with interaction term, Table S6C).

### Number of hatchlings

Hatching success showed a slight increase with laying date (regression coefficient = 0.022 ± 0.005 increase in hatching success per day, Table S9F, Table S6F) and date of snowmelt (regression coefficient = 0.021 ± 0.003 increase in hatching success per day, Table S9G, Table S6G). The number of hatchlings was positively related to clutch size (regression coefficient = 0.24 ± 0.01), and at the same rate in high and low Arctic sites, declined with laying date (regression coefficient = − 0.040 ± 0.008 hatchling per day, Fig. 5c and Table S9D; model with interaction term colony and laying date contains more parameters than model without and model without is 1.1 times more likely than model with interaction term, Table S6D) and date of snowmelt (regression coefficient = − 0.015 ± 0.004 hatchling per day, Fig. 5d and Table S9E; model without interaction term colony and date of snowmelt is 3.1 times more likely than model with interaction term, Table S6E).

### Nesting success

Nesting success decreased with laying date, but the precise shape of the relationship varied between colonies. In the high Arctic (NSK), nesting success was close to zero in 2 years when all nests were predated by polar bears (2012 and 2014). When excluding these years (see “Methods”), nesting success was higher for nests initiated on earlier dates and differed between the low and the high Arctic (NSK: regression coefficient for laying date = − 0.86 ± 0.92; for laying date squared = 0.002 ± 0.003; KB: regression coefficient for laying date = 0.74 ± 1.99; for laying date squared = − 0.002 ± 0.006, Fig. 5e and Table S9H, model with interaction term colony and laying date is 99.0 times more likely than model without interaction term, Table S6H). Nesting success was affected by date of snowmelt (NSK: regression coefficient = − 0.028 ± 0.013 decrease in nesting success per day; KB: regression coefficient = 0.011 ± 0.040, Table S9I, model with date of snowmelt is 1.7 times more likely than model without, Table S6I). However, this effect appeared to be largely caused by differences between colonies (Fig. 5f) and was no longer present in the best performing models when standardizing variables by subtracting the site-specific means of nesting success (model without date of snowmelt is 10.1 times more likely than model with, Table S6J). When comparing peak date of expected number of hatchlings with average laying dates in the high-Arctic colony NSK, geese nested later than the date of peak expected success. In the low-Arctic colony, geese nested in synchrony with the date of peak expected success (Figure S1).

## Discussion

### Advance of egg laying

Congruent with our hypothesis, we found that in the high Arctic geese started laying eggs well before the date of snowmelt, while geese in the low Arctic produced their eggs close to the date of snowmelt. This confirms the idea that the short summer in the high Arctic causes geese to produce eggs relatively early. Surprisingly, while in the high Arctic the snow melted on average 16 days later compared to the low Arctic, average dates of laying in the three study colonies were inversely related to latitude, with geese in the most northern colony laying eggs earlier than in the southern colony. This is in contrast to the finding that the onset of the birds’ breeding season is later at higher latitudes (Owen 1980). Apparently, in our study species individuals are able to acquire body stores for breeding earlier in the high Arctic than in the low Arctic. This contra-intuitive result may arise from high-Arctic geese drawing more from endogenous body stores for egg production (Hahn et al. 2011), and from benefitting from mosses and woody plants available at the very first start of snowmelt (Prop and de Vries 1993; de Fouw et al. 2016). Such an early surge of food, albeit low-quality, is lacking in the low Arctic, where geese depend entirely on graminoids which appear later in the season (van der Graaf et al. 2004, 2006).

We found that both high- and low-Arctic-breeding barnacle geese advanced egg laying at a lower rate than the advance in date of snowmelt (0.27 days advance in laying date per earlier day of snowmelt). This is in line with our hypothesis on laying dates by geese in the high Arctic, where advancements in breeding are likely constrained by a timely arrival at the breeding grounds (Both and Visser 2001; Lameris et al. 2017b). However, we expected that low-Arctic geese would be able to synchronize egg laying with earlier snowmelt as their migration distance is shorter and the continental migration route might enable the geese to track the recession of snow. Our results suggest that low-Arctic geese may be experiencing similar constraints during migration as high-Arctic geese due to a low correlation between climatic conditions along their route (Kölzsch et al. 2015). Alternatively, the observed slow advancement in laying dates in the low Arctic might follow from a set of optimal decision rules (Visser et al. 2012) with highest fitness benefits associated with the observed laying dates.

### Laying dates, timing of snowmelt and fitness components

We did not find a relation between the date of snowmelt and the number of nests, which we use as a proxy of breeding propensity. While several species of geese show a lower breeding propensity in years with a late onset of spring (Reed et al. 2004; Madsen et al. 2007), this appears not to be the case for barnacle geese. This can potentially be explained as barnacle geese primarily breed in coastal areas, where snow-free patches can be found even in years with a late date of snowmelt.

We found a seasonal decline in clutch size and number of hatchlings in both high- and low-Arctic colonies, meaning that early-laying birds produced more eggs and hatchlings. We also found that high- and low-Arctic geese produced larger clutches in earlier springs (a difference of on average 0.6–1.2 eggs between the earliest and latest snowmelt years) and produced more hatchlings (a difference of on average 0.5–0.6 hatchlings between the earliest and latest snowmelt years). A seasonal decline in clutch size is common in geese and birds in general (Drent and Daan 1980; Crick et al. 1993; Rowe et al. 1994; Dalhaug et al. 1996), just as smaller clutches in later springs (Barry 1962; Dalhaug et al. 1996; Bêty et al. 2003; Van Oudenhove et al. 2014).

We found nesting success to be strongly associated with laying date, with a higher probability of hatching at intermediate laying dates in the low Arctic and at early laying dates in the high Arctic, without an additional effect of date of snowmelt. Nest failure in Arctic geese is usually attributed to depletion of body stores by the incubating females (Prop et al. 1984), or to nest predation, such as by glaucous gulls (van der Jeugd et al. 2003), Arctic foxes *Vulpes lagopus* (Jensen et al. 2014) and polar bears (Prop et al. 2015). Geese settling early and well before the peak in laying dates may experience intense predation pressure, as predators are focusing on the few early nests (Findlay and Cooke 1982). Our finding that early-initiated nests in the highArctic are the most successful is in contrast with previous studies, which showed that early breeding geese experienced low nesting success compared to geese starting at intermediate dates (Prop and de Vries 1993; Spaans et al. 2007). This shift is likely caused by polar bears recently moving towards land under climate warming (Iverson et al. 2014; Prop et al. 2015) and predating substantial numbers of nests in bird colonies (Rockwell et al. 2011; Prop et al. 2013, 2015). Our observations indicate that early-initiated nests have a chance to hatch before polar bear arrival and thus may escape predation. This implies that climate warming does not only directly drive reproduction phenology by timing of snowmelt, but also indirectly via changes in predator community composition (Descamps et al. 2017).

Considering our measures of reproductive output, low-Arctic geese lay their eggs at the time of the peak in expected reproductive output (see also Figure S1, van der Jeugd et al. 2009). This suggests that the observed slow advancement in laying dates of low-Arctic geese does not reflect a constraint, but is in line with maximum reproduction output, at least up to the period of hatching. If this is the case, the observed laying dates of low-Arctic geese may be explained as the importance of beneficial conditions for the clutch outweighing those for hatched offspring in years with earlier snowmelt. In contrast, most high-Arctic geese lay their eggs after the expected peak in reproductive output. This suggests that in line with our expectations, high-Arctic geese face a constraint that limits a stronger advancement of laying dates or changes have been too rapid for them to adjust their migration phenology.

## Conclusions

From the moment onwards that the heterogeneity of climate warming effects has been recognized (Gilg et al. 2012), high-Arctic communities are supposed to be especially vulnerable to climate warming (Høye et al. 2007; Post et al. 2009) and are expected to show strong advancements in phenology to counter any negative impacts. We show that both in the high- and low-Arctic, barnacle geese do not advance date of egg laying in pace with earlier dates of snowmelt. However, only in the high Arctic, this advancement appears to be insufficient to reach the level of reproductive success associated with earlier laying dates. For high-Arctic geese, an advance in laying dates may be particularly constrained by the timing of migration, which is thought to be tuned to climate conditions en route rather than to the weather in the Arctic (Tombre et al. 2008; Kölzsch et al. 2015). Given the potential risks of fitness reductions due to phenological mismatches under relatively slow advancement of laying dates (Lameris et al. 2018), high-Arctic bird populations in particular may be prone to negative effects of climate warming.

## Electronic supplementary material

Below is the link to the electronic supplementary material.

Supplementary material 1 (DOCX 132 kb)
