# Supplementary material for: Climate warming may affect the optimal timing of reproduction for migratory geese differently in the low and high Arctic
Source: Oecologia. 2019 Oct 17;191(4):1003–14. doi: 10.1007/s00442-019-04533-7 (PMC6853861; doi:10.1007/s00442-019-04533-7)
Supplement: Supplementary file 1 — Supplementary material 1 (DOCX 132 kb) [file 442_2019_4533_MOESM1_ESM.docx]

**Supplemental material**


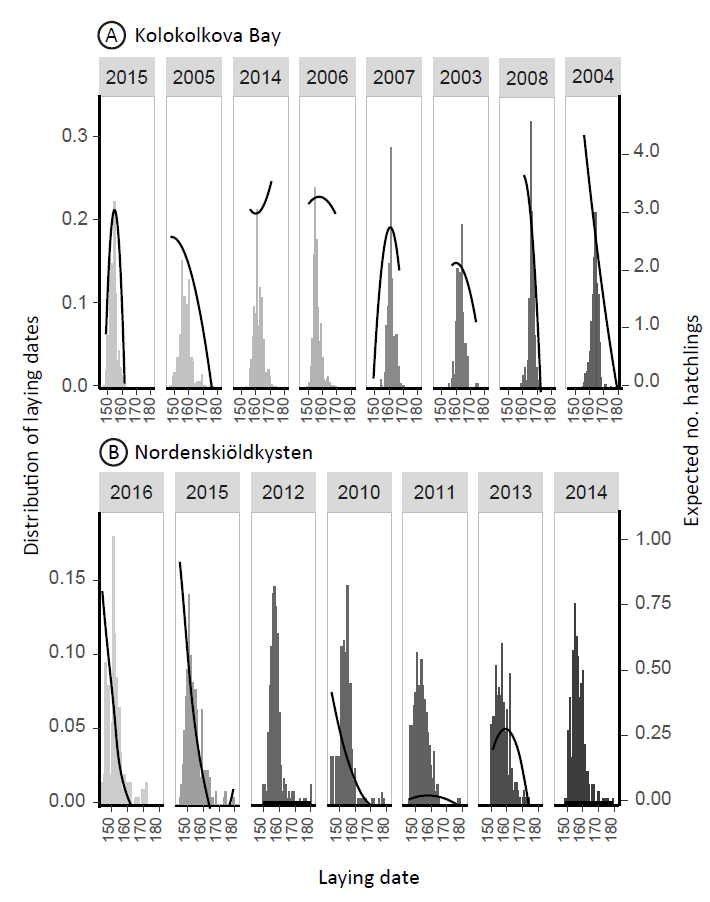


**Figure S1**: Distribution of laying dates (bars) in Kolokolkova Bay and Nordenskiöldysten by year. The timing of snow melt is indicated by intensity of shading of the bars (dark grey – late snowmelt, light grey – early snowmelt), and years are ordered from early to late snowmelt (from left to right). Black lines are based on linear models of expected number of hatchlings, conducted per year (Table S9K).

**Table S1**: Pre-breeding sites and colonies for which snow cover data was determined, including size of the sites in pixels as used for MODIS and in km^2^. As snow cover could not be estimated for the island of Storholmen and Diabasøya, we used adjacent sites where geese were foraging during the laying period.

| **Area** | **Area type** | **Number of pixels (500m^2^)** | **Surface area (km^2^)** | **Latitude** | **Longitude** |
| --- | --- | --- | --- | --- | --- |
| Kolokolkova Bay (KB) | Breeding | 168 | 42 | 68°35'51.60"N | 52°20'12.45"E |
| Nordenskiöldkysten (NSK) | Breeding | 361 | 90.25 | 77°47'18.59"N | 14° 5'51.05"E |
| Kongsfjorden (KF) | Breeding | 64 | 16 | 78°57'42.19"N | 12° 4'9.87"E |
|  |  |  |  | 78°50'57.97"N | 11°50'19.14"E |
| Molotsnii delta (MD) | Staging | 320 | 80 | 68°30'1.49"N | 52° 0'58.65"E |
| Neruta delta (ND) | Staging | 370 | 92.5 | 68°17'4.35"N | 52°16'39.79"E |
| Hornsundneset (HN) | Staging | 78 | 19.5 | 76°52'1.13"N | 15°34'56.68"E |
| Ralstrånda (RS) | Staging | 83 | 20.75 | 77° 1'24.45"N | 15°11'35.85"E |
| Lognedalsflya (LF) | Staging | 90 | 22.5 | 77°30'4.56"N | 13°56'5.96"E |
| Vårsolbukta (VB) | Staging | 73 | 18.25 | 77°46'2.70"N | 14°15'18.91"E |
| Daudmannsøyra (DØ) | Staging | 113 | 28.25 | 78°12'44.16"N | 13°25'44.25"E |
| Sarsøyra (SØ) | Staging | 84 | 21 | 78°45'23.00"N | 11°40'10.35"E |

**Table S2**: Number of nests for which data was available on laying date, nest fate, clutch size and number of hatchlings, grouped per colony and year.

| Colony | Year | Laying date | Nest fate | Clutch size | Hatchlings |
| --- | --- | --- | --- | --- | --- |
| NSK | 2010 | 336 | 336 |  | 19 |
| NSK | 2011 | 225 | 225 |  | 4 |
| NSK | 2012 | 317 | 317 |  |  |
| NSK | 2013 | 226 | 226 |  | 12 |
| NSK | 2014 | 289 | 289 |  |  |
| NSK | 2015 | 225 | 225 |  | 17 |
| NSK | 2016 | 236 | 236 |  | 22 |
| KF | 2000 | 158 |  | 131 | 46 |
| KF | 2001 | 282 |  | 246 | 159 |
| KF | 2003 | 113 |  | 23 |  |
| KF | 2005 | 25 |  | 25 | 21 |
| KF | 2006 | 73 |  | 71 | 42 |
| KF | 2007 | 71 |  | 73 | 32 |
| KF | 2008 | 128 |  | 116 | 83 |
| KF | 2009 | 142 |  | 135 | 95 |
| KF | 2010 | 170 |  | 156 | 102 |
| KF | 2011 | 172 |  | 126 | 73 |
| KF | 2012 | 214 |  | 212 | 119 |
| KF | 2013 | 136 |  | 107 | 54 |
| KF | 2014 | 220 |  | 195 | 118 |
| KF | 2015 | 295 |  | 260 | 130 |
| KF | 2016 | 312 |  | 274 | 170 |
| KB | 2003 | 153 | 153 | 76 | 37 |
| KB | 2004 | 355 | 355 | 220 | 161 |
| KB | 2005 | 344 | 344 | 170 | 86 |
| KB | 2006 | 553 | 553 | 404 | 147 |
| KB | 2007 | 316 | 316 | 122 | 85 |
| KB | 2008 | 446 | 446 |  | 109 |
| KB | 2009 | 698 |  |  |  |
| KB | 2014 | 450 | 450 | 450 | 252 |
| KB | 2015 | 380 | 380 | 276 | 114 |

**Table S3**: Mean day number (number of days from 1 January) of snowmelt and snowfall (± SD), and mean season length in days (± SD), and change in snowmelt / snowfall / season length over time (± SD) for the southern and proximate staging sites (related to breeding colonies) and the breeding colonies themselves.

|  |  | Southern staging site | | Proximate staging site | | Breeding colony | |
| --- | --- | --- | --- | --- | --- | --- | --- |
|  | Colony | Mean date / length | Change, days per year | Mean date / length | Change, days per year | Mean date / length | Change, days per year |
| Snowmelt | KF | 160 ± 7 | -0.47 ± 0.32 | 169 ± 9 | -0.76 ± 0.44 | 167 ± 9 | –0.67 ± 0.41 |
|  | NSK | 160 ± 7 | -0.47 ± 0.32 | 163 ± 9 | -0.75 ± 1.06 | 168 ± 8 | –0.53 ± 1.00 |
|  | KB |  |  | 148 ± 9 | -0.60 ± 1.06 | 155 ± 9 | –0.85 ± 1.00 |
| Snowfall | KF | 259 ± 7 | 0.15 ± 0.37 | 244 ± 10 | -0.01 ± 0.51 | 248 ± 9 | 1.05 ± 0.34 |
|  | NSK | 259 ± 7 | 0.15 ± 0.37 | 254 ± 11 | 0.62 ± 0.38 | 252 ± 11 | 0.57 ± 0.54 |
|  | KB |  |  | 261 ± 12 | 0.12 ± 0.41 | 252 ± 10 | 0.96 ± 0.46 |
| Season length | KF | 99 ± 11 | 0.61 ± 0.53 | 75 ± 12 | 0.75 ± 0.61 | 81 ± 12 | 1.73 ± 0.64 |
|  | NSK | 99 ± 11 | 0.61 ± 0.53 | 91 ± 14 | 1.38 ± 1.47 | 84 ± 13 | 1.11 ± 1.56 |
|  | KB |  |  | 113 ± 12 | 0.72 ± 1.47 | 97 ± 14 | 1.80 ± 1.56 |

**Table S4**: Linear mixed effect models (LMMs) for date of snowmelt (SM), date of snowfall (SF) and season length (SL). Models include fixed effects area (A), site type (ST) and year (Y. Included random effects are year ((Y)) and / or site ((S)). Models are ordered from lowest to highest AICc values, models included in model averaging are marked **bold**. Reported R^2^ is the conditional R^2^.

| **Model** | **A) Snowmelt (SM)** | | Degrees of freedom | AIC_c_ | Δ AIC_c_ | Model weight | R^2^ | |
| --- | --- | --- | --- | --- | --- | --- | --- | --- |
| **1** | **SM ~ A + Y + (S)** | | 5 | 1339.7 | 0.0 | 0.74 | 0.42 | |
| 2 | SM ~ A + Y + A*Y + (S) | | 6 | 1341.8 | 2.1 | 0.25 |  | |
| 3 | SM ~ A + (S) | | 4 | 1356.4 | 16.8 | 0.00 |  | |
| 4 | SM ~ Y + (S) | | 4 | 1364.2 | 24.5 | 0.00 |  | |
|  | | | | | | | |  |
|  | **B) Snowmelt (SM)** | | Degrees of freedom | AIC_c_ | Δ AIC_c_ | Model weight | R^2^ | |
| **1** | **SM ~ A + ST + (S) + (Y)** | | 6 | 1259.8 | 0.0 | 0.52 | 0.37 | |
| 2 | SM ~ A + ST + A*ST + (S) + (Y) | | 7 | 1260.4 | 0.6 | 0.38 |  | |
| 3 | SM ~ A + (S) + (Y) | | 5 | 1262.9 | 3.2 | 0.11 |  | |
| 4 | SM ~ ST + (S) + (Y) | | 5 | 1280.5 | 20.7 | 0.00 |  | |
|  |  |  | | | | | |  |
|  | **C) Snowfall (SF)** | | Degrees of freedom | AIC_c_ | Δ AIC_c_ | Model weight | R^2^ | |
| **1** | **SF ~ A + Y + (S)** | | 5 | 1407.5 | 0.0 | 0.47 | 0.08 | |
| **2** | **SF ~ Y + (S)** | | 4 | 1408.2 | 0.7 | 0.33 |  | |
| 3 | SF ~ A + Y + A*Y + (S) | | 6 | 1409.6 | 2.1 | 0.16 |  | |
| 4 | SF ~ A + (S) | | 4 | 1412.7 | 5.2 | 0.04 |  | |
|  |  |  | | | | | |  |
|  | **D) Season Length (SL)** | | Degrees of freedom | AIC_c_ | Δ AIC_c_ | Model weight | R^2^ | |
| **1** | **SL ~ A + Y + (S)** | | 5 | 1523.1 | 0.0 | 0.74 | 0.33 | |
| 2 | SL ~ A + Y + A*Y + (S) | | 6 | 1525.2 | 2.1 | 0.25 |  | |
| 3 | SL ~ Y + (S) | | 4 | 1531.4 | 8.3 | 0.01 |  | |
| 4 | SL ~ A + (S) | | 4 | 1547.8 | 24.7 | 0.00 |  | |

**Table S5**: Linear mixed effect models (LMMs) and generalized linear regression models (GLMs) for laying date (LD). Models include fixed effects colony (C), year (Y), date of snowmelt in colonies (SM) and date of snowmelt on proximate staging sites (SMS). The included random effect is year ((Y)). Models are ordered from lowest to highest AICc values, models included in model averaging are marked **bold**. Reported R^2^ is the adjusted R^2^.

| **Model** | **A) Laying date (LD) ~ year (Y)** | | | Degrees of freedom | AIC_c_ | Δ AIC_c_ | Model weight | R^2^ |
| --- | --- | --- | --- | --- | --- | --- | --- | --- |
| **1** | **LD ~ C + Y** | | | 5 | 163.4 | 0.0 | 0.84 | 0.63* |
| 2 | LD ~ C + Y + C*Y | | | 7 | 166.9 | 3.5 | 0.15 |  |
| 3 | LD ~ C | | | 4 | 172.0 | 8.6 | 0.01 |  |
| 4 | LD ~ Y | | | 3 | 186.4 | 23.0 | 0.00 |  |
|  |  |  |  |  |  |  |  |  |
|  | **B) Laying date (LD) ~ Snowmelt (SM)** | | | Degrees of freedom | AIC_c_ | Δ AIC_c_ | Model weight | R^2^ |
| **1** | **LD ~ SMS + C + (Y)** | | | 6 | 149.3 | 0.0 | 0.56 | 0.81 |
| 2 | LD ~ SMS + C + SMS*C + (Y) | | | 8 | 150.5 | 1.2 | 0.31 |  |
| 3 | LD ~ SM + SMS + C + (Y) | | | 7 | 152.6 | 3.3 | 0.11 |  |
| 4 | LD ~ SM + SMS + SM*C + (Y) | | | 9 | 156.1 | 6.8 | 0.02 |  |
| 5 | LD ~ SM + C + (Y) | | | 6 | 157.8 | 8.5 | 0.01 |  |
| 6 | LD ~ SM + C + SM*C + (Y) | | | 8 | 161.2 | 11.9 | 0.00 |  |
| 7 | LD ~ SM + SMS + SM*C + SMS*C + (Y) | | | 11 | 163.7 | 14.4 | 0.00 |  |
| 8 | LD ~ C | | | 5 | 169.0 | 19.7 | 0.00 |  |
| 9 | LD ~ SM + SMS | | | 5 | 190.2 | 40.9 | 0.00 |  |
| 10 | LD ~ SM | | | 4 | 193.0 | 43.7 | 0.00 |  |
| 11 | LD ~ SMS | | | 4 | 194.4 | 45.1 | 0.00 |  |

* Marginal R^2^.

**Table S6**: Generalized linear regression models (GLMs) for amount of nests (NN), total clutch size (TC), total number of hatchlings (TH), hatching success (the proportion of eggs that hatched; HS) and nesting success (NS). Models include fixed effects colony (C), date of snowmelt on proximate staging sites (SMS) and laying date (LD). Models are ordered from lowest to highest AICc values, models included in model averaging are marked **bold**. Reported R^2^ is the adjusted R^2^.

| **Model** | **A) Amount of nests (NN)** | degrees of freedom | AIC_c_ | Δ AIC_c_ | Model weight | R^2^ |
| --- | --- | --- | --- | --- | --- | --- |
| **1** | **NN ~ C** | 4 | 360.9 | 0.0 | 0.81 | 0.47 |
| 2 | NN ~ C + SM | 5 | 363.8 | 2.9 | 0.19 |  |
| 3 | NN ~ SM | 3 | 373.1 | 12.2 | 0.00 |  |
|  |  | | | | | |
|  | **B) Total clutch size (TC) ~ laying date (LD)** | degrees of freedom | AIC_c_ | Δ AIC_c_ | Model weight | R^2^ |
| **1** | **TC ~ C + LD + C*LD** | 4 | 13263.7 | 0.0 | 0.99 | 0.11 |
| 2 | TC ~ C + LD | 3 | 13273.2 | 9.6 | 0.01 |  |
| 3 | TC ~ C | 2 | 13335.4 | 71.8 | 0.00 |  |
| 4 | TC ~ LD | 2 | 13357.8 | 94.2 | 0.00 |  |
|  | **C) Total clutch size (TC) ~ snowmelt staging (SMS)** | degrees of freedom | AIC_c_ | Δ AIC_c_ | Model weight | R^2^ |
| **1** | **TC ~ C + SMS** | 3 | 14603.9 | 0.0 | 0.58 | 0.12 |
| 2 | TC ~ C + SMS + C*SMS | 4 | 14605.3 | 1.4 | 0.29 |  |
| 3 | TC ~ SMS | 2 | 14606.8 | 2.9 | 0.13 |  |
| 4 | TC ~ C | 2 | 14697.8 | 93.9 | 0.00 |  |
|  |  | | | | | |
|  | **D) Total number of hatchlings (TH) ~ laying date (LD)** | degrees of freedom | AIC_c_ | Δ AIC_c_ | Model weight | R^2^ |
| **1** | **TC ~ C + LD** | 4 | 7876.6 | 0.0 | 0.52 | 0.05 |
| 2 | TC ~ C + LD + C*LD | 6 | 7876.8 | 0.2 | 0.48 |  |
| 3 | TC ~ C | 3 | 7899.4 | 22.8 | 0.00 |  |
| 4 | TC ~ LD | 2 | 7912.5 | 35.9 | 0.00 |  |
|  | **E) Total number of hatchlings (TH) ~ snowmelt staging (SMS)** | degrees of freedom | AIC_c_ | Δ AIC_c_ | Model weight | R^2^ |
| **1** | **TH ~ C + SMS** | 4 | 7893.7 | 0.0 | 0.67 | 0.03 |
| 2 | TH ~ SMS | 2 | 7896.0 | 2.3 | 0.21 |  |
| 3 | TH ~ C + SMS + C*SMS | 6 | 7897.0 | 3.4 | 0.12 |  |
| 4 | TH ~ C | 3 | 7905.5 | 11.9 | 0.00 |  |
|  |  | | | | | |
|  | **F) Hatching success (HS) ~ laying date (LD)** | degrees of freedom | AIC_c_ | Δ AIC_c_ | Model weight | R^2^ |
| **1** | **HS ~ LD** | 2 | 4021.7 | 0.0 | 0.55 | 0.01 |
| 2 | HS ~ C + LD | 3 | 4023.4 | 1.6 | 0.25 |  |
| 3 | HS ~ C + LD + C*LD | 4 | 4023.9 | 2.1 | 0.19 |  |
| 4 | HS ~ C | 2 | 4029.7 | 7.9 | 0.01 |  |
|  | **G) Hatching success (HS) ~ snowmelt staging (SMS)** | degrees of freedom | AIC_c_ | Δ AIC_c_ | Model weight | R^2^ |
| **1** | **HS ~ C + SMS** | 3 | 3993.6 | 0.0 | 0.69 | 0.02 |
| 2 | HS ~ C + SMS + C*SMS | 4 | 3995.1 | 1.6 | 0.31 |  |
| 3 | HS ~ C | 2 | 4029.7 | 36.1 | 0.00 |  |
| 4 | HS ~ SMS | 2 | 4034.0 | 40.5 | 0.00 |  |
|  |  | | | | | |
|  | **H) Nesting success (NS) ~ laying date (LD)** | degrees of freedom | AIC_c_ | Δ AIC_c_ | Model weight | R^2^ |
| **1** | **NS ~ LD + LD^2 + C + LD*C + LD^2*C** | 6 | 929.2 | 0.0 | 0.99 | 0.82 |
| 2 | NS ~ LD + LD^2 + C | 4 | 939.9 | 10.6 | 0.01 |  |
| 3 | NS ~ LD + C | 3 | 940.7 | 11.5 | 0.00 |  |
| 4 | NS ~ C | 2 | 955.4 | 26.2 | 0.00 |  |
|  | **I) Nesting success (NS) ~ snowmelt staging (SMS)** | degrees of freedom | AIC_c_ | Δ AIC_c_ | Model weight | R^2^ |
| **1** | **NS ~ C + SMS + C*SMS** | 4 | 358.6 | 0.0 | 0.56 | 0.86 |
| **2** | **NS ~ C** | 2 | 359.7 | 1.1 | 0.33 |  |
| 3 | NS ~ C + SMS | 3 | 361.9 | 3.2 | 0.11 |  |
| 4 | NS ~ SMS | 2 | 1977.2 | 1618.6 | 0.00 |  |
|  | **J) Scaled nesting success (SNS) ~ snowmelt staging (SMS)** | degrees of freedom | AIC_c_ | Δ AIC_c_ | Model weight | R^2^ |
| **1** | **SNS ~ C** | 3 | 12.1 | 0.0 | 0.91 | 0.91 |
| 2 | SNS ~ C + SMS | 4 | 16.7 | 4.6 | 0.09 |  |
| 3 | SNS ~ C + SMS + C*SMS | 5 | 22.4 | 10.3 | 0.00 |  |
| 4 | SNS ~ SMS | 3 | 40.4 | 28.3 | 0.00 |  |
|  | **K) Total expected hatchlings (TEH) ~ laying date (LD)** | degrees of freedom | AIC_c_ | Δ AIC_c_ | Model weight | R^2^ |
| **1** | **TEH ~ LD + LD^2 + C + LD*S + LD^2*C + (Y)** | 8 | 13811.5 | 0.0 | 1.00 | 0.53* |
| 2 | TEH ~ LD + C + (Y) | 5 | 13832.0 | 20.5 | 0.00 |  |
| 3 | TEH ~ LD + LD^2 + C + (Y) | 6 | 13846.5 | 35.0 | 0.00 |  |
| 4 | TEH ~ C + (Y) | 4 | 13915.7 | 104.2 | 0.00 |  |
| 5 | TEH ~ LD + (Y) | 4 | 15348.1 | 1536.6 | 0.00 |  |

* Marginal R^2^.

**Table S7**: Model averaging results for coefficient values (mean ± standard deviation) for generalized linear regression models (GLMs) reported in Table S4.

| **A) Snowmelt ~ area + year + (site)** | intercept | year |
| --- | --- | --- |
| high Arctic | 1476.6 ± 247.4 | -0.66 ± 0.12 |
| low Arctic | 1491.2 ± 249.5 | -0.66 ± 0.12 |
|  | | |
| **B) Snowmelt ~ area + site type** | intercept |  |
| high Arctic - colony | 168.4 ± 4.0 |  |
| high Arctic - staging site | 163.9 ± 5.7 |  |
| low Arctic - colony | 153.3±2.4 |  |
| low Arctic - staging site | 148.9 ± 5.7 |  |
|  |  |  |
| **C) Snowfall ~ area + year** | intercept | year |
| high Arctic | -548.6 ± 236.1 | 0.40 ± 0.15 |
| low Arctic | -545.4 ± 293.1 | 0.40 ± 0.15 |
|  |  |  |
| **D) Season length ~ area + year** | intercept | year |
| high Arctic | -2040.3 ± 400.0 | 1.06 ± 0.20 |
| low Arctic | -2020.3 ± 395.2 | 1.06 ± 0.20 |

**Table S8**: Model averaging results for coefficient values (mean ± standard deviation) for linear mixed effect models (LMMs) and generalized linear regression models (GLMs) reported in Table S5.

| **A) Laying date ~ year + colony** | intercept | year |
| --- | --- | --- |
| KF | 1012.3 ± 247.5 | -0.43 ± 12 |
| NSK | 1016.9 ± 1.4 | -0.43 ± 12 |
| KB | 1020.2 ± 1.3 | -0.43 ± 12 |
|  | | |
| **B) Laying date ~ snowmelt staging + colony** | intercept | snowmelt staging |
| KF | 109.4 ± 8.5 | 0.27 ± 0.04 |
| NSK | 121.3 ± 19.9 | 0.27 ± 0.04 |
| KB | 113.7 ± 22.2 | 0.27 ± 0.04 |

**Table S9**: Model averaging results for coefficient values (mean ± standard deviation) for linear mixed effect models (LMMs) reported in Table S6.

| **A) Amount of nests ~ colony** | intercept |  |  |  |
| --- | --- | --- | --- | --- |
| KF | 170.3 ± 23.0 |  |  |  |
| NSK | 219.4 ± 63.74 |  |  |  |
| KB | 374.6 ± 62.0 |  |  |  |
|  |  |  |  |  |
| **B) Total clutch size ~ colony + laying date + colony*laying date** | intercept | laying date | snowmelt staging |  |
| KF | 9.39 ± 1.22 | -0.037 ± 0.008 |  |  |
| KB | 17.44 ± 3.39 | -0.084 ± 0.020 |  |  |
| **C) Total clutch size ~ colony + snowmelt staging** |  |  |  |  |
| KF | 9.36 ± 0.64 |  | -0.034 ± 0.004 |  |
| KB | 8.82 ± 1.49 |  | -0.034 ± 0.004 |  |
|  |  |  |  |  |
| **D) Total hatchlings ~ colony + laying date** | intercept | laying date | snowmelt staging |  |
| KF | 8.52 ± 1.55 | -0.040 ± 0.008 |  |  |
| NSK | 6.09 ± 6.84 | -0.040 ± 0.008 |  |  |
| KB | 11.34 ± 4.52 | -0.040 ± 0.008 |  |  |
| **E) Total hatchlings ~ colony + snowmelt staging** |  |  |  |  |
| KF | 5.72 ± 0.69 |  | -0.015 ± 0.004 |  |
| NSK | 5.19 ± 0.90 |  | -0.015 ± 0.004 |  |
| KB | 5.72 ± 0.80 |  | -0.015 ± 0.004 |  |
|  |  |  |  |  |
| **F) Hatching success ~ colony + laying date** | intercept | laying date | snowmelt staging |  |
| KF | -1.70 ± 0.92 | 0.022 ± 0.005 |  |  |
| KB | -1.68 ± 0.97 | 0.022 ± 0.005 |  |  |
| **G) Hatching success ~ colony + snowmelt staging** |  |  |  |  |
| KF | -1.96 ± 0.59 |  | 0.021 ± 0.003 |  |
| KB | -1.11 ± 1.39 |  | 0.021 ± 0.003 |  |
|  |  |  |  |  |
| **H) Nesting success ~ laying date + laying date^2 + colony + laying date*colony + laying date^2*colony** | intercept | laying date | laying date ^2 | snowmelt staging |
| NSK | 73.27 ± 70.73 | -0.86 ± 0.92 | 0.002 ± 0.003 |  |
| KB | -56.23 ± 155.14 | 0.74 ± 1.99 | -0.002 ± 0.006 |  |
| **I) Nesting success ~ colony + snowmelt staging + colony*snowmelt staging** |  |  |  |  |
| NSK | 0.47 ± 2.69 |  |  | -0.028 ± 0.013 |
| KB | 0.43 ± 6.11 |  |  | 0.011 ± 0.040 |
| **J) Scaled nesting success ~ colony** |  |  |  |  |
| NSK | -1.09 ± 0.13 |  |  |  |
| KB | 0.78 ± 0.31 |  |  |  |
| **K) Total expected hatchlings ~ laying date + laying date^2 + colony + laying date*colony + laying date^2*colony** | intercept | laying date | laying date ^2 |  |
| NSK | 42.40 ± 13.50 | -0.51 ± 0.17 | 0.002 ± 0.001 |  |
| KB | -85.04 ± 38.36 | 1.17 ± 0.48 | -0.004 ± 0.002 |  |
